# Comparison of short-term outcomes from the International Oesophago-Gastric Anastomosis Audit (OGAA), the Esophagectomy Complications Consensus Group (ECCG), and the Dutch Upper Gastrointestinal Cancer Audit (DUCA)

**DOI:** 10.1093/bjsopen/zrab010

**Published:** 2021-05-22

**Authors:** Oesophago-Gastric Anastomosis Study Group, J Fergusson, E Beenen, C Mosse, J Salim, S Cheah, T Wright, MP Cerdeira, P McQuillan, M Richardson, H Liem, J Spillane, M Yacob, F Albadawi, T Thorpe, A Dingle, C Cabalag, K Loi, OM Fisher, S Ward, M Read, M Johnson, R Bassari, H Bui, I Cecconello, RAA Sallum, JRM da Rocha, LR Lopes, V Tercioti, JDS Coelho, JAP Ferrer, G Buduhan, L Tan, S Srinathan, P Shea, J Yeung, F Allison, P Carroll, F Vargas-Barato, F Gonzalez, J Ortega, L Nino-Torres, TC Beltrán-García, L Castilla, M Pineda, A Bastidas, J Gómez-Mayorga, N Cortés, C Cetares, S Caceres, S Duarte, A Pazdro, M Snajdauf, H Faltova, M Sevcikova, PB Mortensen, N Katballe, T Ingemann, B Morten, I Kruhlikava, AP Ainswort, NM Stilling, J Eckardt, J Holm, M Thorsteinsson, M Siemsen, B Brandt, B Nega, E Teferra, A Tizazu, JS Kauppila, V Koivukangas, S Meriläinen, R Gruetzmann, C Krautz, G Weber, H Golcher, G Emons, A Azizian, M Ebeling, S Niebisch, N Kreuser, G Albanese, J Hesse, L Volovnik, U Boecher, M Reeh, S Triantafyllou, D Schizas, A Michalinos, E Mpali, M Mpoura, A Charalabopoulos, DK Manatakis, D Balalis, J Bolger, C Baban, A Mastrosimone, O McAnena, A Quinn, CB Ó Súilleabháin, MM Hennessy, I Ivanovski, H Khizer, N Ravi, N Donlon, M Cervellera, S Vaccari, S Bianchini, l Sartarelli, E Asti, D Bernardi, S Merigliano, L Provenzano, M Scarpa, L Saadeh, B Salmaso, G De Manzoni, S Giacopuzzi, R La Mendola, CA De Pasqual, Y Tsubosa, M Niihara, T Irino, R Makuuchi, K Ishii, M Mwachiro, A Fekadu, A Odera, E Mwachiro, D AlShehab, HA Ahmed, AO Shebani, A Elhadi, FA Elnagar, HF Elnagar, ST Makkai-Popa, LF Wong, T Yunrong, S Thanninalai, HC Aik, PW Soon, TJ Huei, HNL Basave, R Cortés-González, SM Lagarde, JJB van Lanschot, C Cords, WA Jansen, I Martijnse, R Matthijsen, S Bouwense, B Klarenbeek, M Verstegen, F van Workum, JP Ruurda, PC van der Sluis, M de Maat, N Evenett, P Johnston, R Patel, A MacCormick, M Young, B Smith, C Ekwunife, AH Memon, K Shaikh, A Wajid, N Khalil, M Haris, ZU Mirza, SBA Qudus, MZ Sarwar, A Shehzadi, A Raza, MH Jhanzaib, J Farmanali, Z Zakir, O Shakeel, I Nasir, S Khattak, M Baig, MA Noor, HH Ahmed, A Naeem, AC Pinho, R da Silva, H Matos, T Braga, C Monteiro, P Ramos, F Cabral, MP Gomes, PC Martins, AM Correia, JF Videira, C Ciuce, R Drasovean, R Apostu, C Ciuce, S Paitici, AE Racu, CV Obleaga, M Beuran, B Stoica, C Ciubotaru, V Negoita, I Cordos, RD Birla, D Predescu, PA Hoara, R Tomsa, V Shneider, M Agasiev, I Ganjara, D Gunjic´, M Veselinovic´, T Babič, TS Chin, A Shabbir, G Kim, A Crnjac, H Samo, I Díez del Val, S Leturio, I Díez del Val, S Leturio, JM Ramón, M Dal Cero, S Rifá, M Rico, A Pagan Pomar, JA Martinez Corcoles, JL Rodicio Miravalles, SA Pais, SA Turienzo, LS Alvarez, PV Campos, AG Rendo, SS García, EPG Santos, ET Martínez, MJ Fernández Díaz, C Magadán Álvarez, V Concepción Martín, C Díaz López, A Rosat Rodrigo, LE Pérez Sánchez, M Bailón Cuadrado, C Tinoco Carrasco, E Choolani Bhojwani, DP Sánchez, ME Ahmed, T Dzhendov, F Lindberg, M Rutegård, M Sundbom, C Mickael, N Colucci, A Schnider, S Er, E Kurnaz, S Turkyilmaz, A Turkyilmaz, R Yildirim, BE Baki, N Akkapulu, O Karahan, N Damburaci, R Hardwick, P Safranek, V Sujendran, J Bennett, Z Afzal, M Shrotri, B Chan, K Exarchou, T Gilbert, T Amalesh, D Mukherjee, S Mukherjee, TH Wiggins, R Kennedy, S McCain, A Harris, G Dobson, N Davies, I Wilson, D Mayo, D Bennett, R Young, P Manby, N Blencowe, M Schiller, B Byrne, D Mitton, V Wong, A Elshaer, M Cowen, V Menon, LC Tan, E McLaughlin, R Koshy, C Sharp, H Brewer, N Das, M Cox, W Al Khyatt, D Worku, R Iqbal, L Walls, R McGregor, G Fullarton, A Macdonald, C MacKay, C Craig, S Dwerryhouse, S Hornby, S Jaunoo, M Wadley, C Baker, M Saad, M Kelly, A Davies, F Di Maggio, S McKay, P Mistry, R Singhal, O Tucker, S Kapoulas, S Powell-Brett, P Davis, G Bromley, L Watson, R Verma, J Ward, V Shetty, C Ball, K Pursnani, A Sarela, H Sue Ling, S Mehta, J Hayden, N To, T Palser, D Hunter, K Supramaniam, Z Butt, A Ahmed, S Kumar, A Chaudry, O Moussa, A Kordzadeh, B Lorenzi, J Willem, G Bouras, R Evans, M Singh, H Warrilow, A Ahmad, N Tewari, F Yanni, J Couch, E Theophilidou, JJ Reilly, P Singh, Gijs van Boxel, K Akbari, D Zanotti, B Sgromo, G Sanders, T Wheatley, A Ariyarathenam, A Reece-Smith, L Humphreys, C Choh, N Carter, B Knight, P Pucher, A Athanasiou, I Mohamed, B Tan, M Abdulrahman, J Vickers, K Akhtar, R Chaparala, R Brown, MMA Alasmar, R Ackroyd, K Patel, A Tamhankar, A Wyman, R Walker, B Grace, N Abbassi, N Slim, L Ioannidi, G Blackshaw, T Havard, X Escofet, A Powell, A Owera, F Rashid, P Jambulingam, J Padickakudi, H Ben-Younes, K Mccormack, IA Makey, MK Karush, CW Seder, MJ Liptay, G Chmielewski, EL Rosato, AC Berger, R Zheng, E Okolo, A Singh, CD Scott, MJ Weyant, JD Mitchell

## Abstract

**Background:**

The Esophagectomy Complications Consensus Group (ECCG) and the Dutch Upper Gastrointestinal Cancer Audit (DUCA) have set standards in reporting outcomes after oesophagectomy. Reporting outcomes from selected high-volume centres or centralized national cancer programmes may not, however, be reflective of the true global prevalence of complications. This study aimed to compare complication rates after oesophagectomy from these existing sources with those of an unselected international cohort from the Oesophago-Gastric Anastomosis Audit (OGAA).

**Methods:**

The OGAA was a prospective multicentre cohort study coordinated by the West Midlands Research Collaborative, and included patients undergoing oesophagectomy for oesophageal cancer between April and December 2018, with 90 days of follow-up.

**Results:**

The OGAA study included 2247 oesophagectomies across 137 hospitals in 41 countries. Comparisons with the ECCG and DUCA found differences in baseline demographics between the three cohorts, including age, ASA grade, and rates of chronic pulmonary disease. The OGAA had the lowest rates of neoadjuvant treatment (OGAA 75.1 per cent, ECCG 78.9 per cent, DUCA 93.5 per cent; *P* < 0.001). DUCA exhibited the highest rates of minimally invasive surgery (OGAA 57.2 per cent, ECCG 47.9 per cent, DUCA 85.8 per cent; *P* < 0.001). Overall complication rates were similar in the three cohorts (OGAA 63.6 per cent, ECCG 59.0 per cent, DUCA 62.2 per cent), with no statistically significant difference in Clavien–Dindo grades (*P* = 0.752). However, a significant difference in 30-day mortality was observed, with DUCA reporting the lowest rate (OGAA 3.2 per cent, ECCG 2.4 per cent, DUCA 1.7 per cent; *P* = 0.013).

**Conclusion:**

Despite differences in rates of co-morbidities, oncological treatment strategies, and access to minimal-access surgery, overall complication rates were similar in the three cohorts.

## Teaser

The Oesophago-Gastric Anastomosis Audit (OGAA) was a prospective multicentre cohort study which included 2247 oesophagectomies across 137 hospitals in 41 countries. The article compares the rates of complications after oesophagectomy from the Esophagectomy Complications Consensus Group, the Dutch Upper Gastrointestinal Cancer Audit, and the OGAA to provide a comprehensive assessment of the current standard of global oesophageal surgery.

## Introduction

Oesophageal cancer is a major cause of global mortality, accounting for more than 436 000 deaths annually^1^. Late presentation frequently means that only 30–40 per cent of patients are suitable for curative treatment options^2^^,^^3^. Oesophagectomy remains an integral part of the curative treatment in this latter group, but is associated with significant morbidity and mortality^4^^,^^5^. Before the establishment of definitions for complications and quality measures by the Esophageal Complications Consensus Group (ECCG) in 2015^6^, it was challenging to evaluate the international variation in postoperative oesophagectomy outcomes. The ECCG has provided postoperative outcomes from 24 selected high-volume centres, setting a benchmark for high-quality oesophageal surgery reporting^4^. The Dutch Upper Gastrointestinal Cancer Audit (DUCA) has also provided detailed outcome data from a nationally centralized oesophageal cancer programme, further highlighting national and international variation in complications^5^.

Patient co-morbidity has a significant impact on postoperative outcomes and plays a critical role in achieving good outcomes^7–9^, whether surgery involves a minimally invasive or open operation^10^^,^^11^.

The Oesophago-Gastric Anastomosis Audit (OGAA)^12^ was undertaken in 2018 to provide a comprehensive assessment of preoperative, intraoperative, and postoperative oesophagectomy outcomes, with a detailed appraisal of complications, in accordance with the ECCG framework. The study aimed to collect data from a large number of centres and countries, encompassing centres with varying levels of experience and patient volumes. Centre inclusion was by open invitation, as opposed to the invitation-only approach used by the international ECCG study and compulsory inclusion in DUCA, in an attempt to include this broader perspective.

The aims of the study were to report comprehensive short-term postoperative outcomes from the OGAA cohort, and to provide detailed comparative analyses against the ECCG and DUCA benchmarking studies.

## Methods

The OGAA study was run by the Oesophago-Gastric Anastomosis Study Group, on behalf of the West Midlands Research Collaborative. Centres performing oesophagectomy for cancer were invited to contribute. There was no minimum unit volume to register for the study and participation was voluntary. Committed surgeons from each country were invited to act as national lead as part of the organizing committee. This permitted language-specific dissemination of study material and advertising, to facilitate wider centre recruitment. Opportunities for voluntary participation were circulated through national surgical societies. A dedicated social media team facilitated global engagement of international oesophageal surgical centres through a number of platforms. All endeavours were made to ensure open and inclusive centre recruitment, to provide a comprehensive global cohort. The protocol for this study has been published^12^^,^^13^ along with the collaborative model that has successfully delivered a number of international and national cohort studies^14–17^.

Centres were not required to standardize surgical or management pathways, and no changes were made to individual aspects of patient care as part of the study. Teams of surgeons, surgical trainees, research nurses or medical students prospectively identified eligible patients over a 9-month period from 2 April 2018 to 31 December 2018. Patients were followed for 90 days from the date of surgical resection, with study follow-up closing on 31 March 2019. Data collection teams at each centre were supervised by a consultant surgeon, who took overall responsibility for local study conduct and data validation. No external data validation was performed on submitted data, in keeping with previously published data by the ECCG^4^. Data submitted to DUCA are subject to external validation to ensure completeness and accuracy.

### Outcome measures

The primary aim was to assess the comparative frequency of postoperative complications (within 30 days) across the OGAA, ECCG, and DUCA cohorts. Complications were defined by the ECCG framework^6^, and classified based on the Clavien–Dindo grade^18^. Secondary outcomes were reoperation, readmission, and postoperative mortality rates. Outcome data for the ECCG and DUCA cohorts were sourced from the most recent publications at the time of conception of the present study. This therefore encompassed patients undergoing oesophagectomy from January 2015 to December 2016 and from January 2016 to December 2017 respectively^4^^,^^5^. Data on oesophagectomy and gastrectomy were reported separately for the DUCA cohort, and only oesophagectomy data were included in the present analysis. As data were acquired from published materials, individual patient-level data were not available, so statistical adjustment for baseline difference between cohorts was not feasible. This also prevented exclusion of patients from ECCG and DUCA cohorts, such as those with benign disease or without an anastomosis

Tumour staging was performed in accordance with the eighth edition of the TNM staging classification^19^. Positive tumour margins in the OGAA were defined as tumour identifiable at 1 mm or less, in accordance with the Royal College of Pathologists guidance^20^. However, positive tumour margins in the ECCG and DUCA studies were defined as tumour identifiable at 0 mm, in accordance with College of American Pathologists guidance^21^. Comparison of margin positivity (R status) between the OGAA and ECCG/DUCA was therefore not possible.

### Ethical approval and data sharing for OGAA

Ethical approval was dependent on local protocols and was country-specific. It was the responsibility of the local principal investigator of the enrolled unit to ensure that appropriate ethical or audit approval was gained before commencement of the study. Ongoing study approval was maintained locally throughout the duration of the study. In the UK, the study was registered at each site as either a clinical audit or service evaluation, on the basis that the information collected was routine and anonymized with no influence on the clinical care pathway.

### Statistical analysis

For variables that were available for all three cohorts, comparisons were done using χ^2^ tests for nominal variables, and Kruskal–Wallis tests for ordinal variables. Where significant differences were detected, *post hoc* pairwise comparisons were performed using χ^2^ tests or Mann–Whitney *U* tests, as applicable, with Bonferroni correction for three comparisons applied to the resulting *P* values. Variables that were reported only for two cohorts were analysed using χ^2^ tests or Mann–Whitney *U* tests, as applicable. All analyses were carried out using SPSS^®^ version 22 (IBM, Armonk, New York, USA), with *P* < 0.050 deemed indicative of statistical significance throughout.

## Results

Between April and December 2018, 2247 oesophagectomies for cancer were included in the OGGA. A summary of the characteristics of this study and those of the ECCG and DUCA cohorts is shown in *Table 1.* The OGAA included patients from 137 centres across 41 countries (106 centres in high-income countries, 31 centres in low–middle-income countries). Of centres contributing to the OGAA, 71 were located in 13 countries where oesophageal adenocarcinoma (OAC) was the predominant histological type^22^. The ECCG and DUCA studies included patients from fewer centres (24 and 22 respectively) in fewer countries (14 and 1 respectively). Fourteen centres contributing to the ECCG were located in six countries with a histological predominance of adenocarcinoma (OAC) over squamous cell carcinoma (SCC). The DUCA encompasses centres only in the Netherlands, where the age-standardized incidence per 100 000 population is 4.4 for OAC and 2.0 for SCC^22^.

**Table 1:** Summary of study characteristics of cohort studies from the OGAA, ECCG and DUCA

	OGAA	ECCG	DUCA
No. of centres	137	24	22
No. of countries	41	14	1
No. of patients	2247	2704	1617
Inclusion dates	Apr 2018 to Dec 2018	Jan 2015 to Dec 2016	Jan 2016 to Dec 2017
Centre volume	Any	High volume only	> 20 resections/year
Centre inclusion	Open invitation (national leads dissemination, national societies, social media)	Invite only (2020— open to new applications)	Mandatory national audit
Type of surgery	Oesophagectomy only	Oesophagectomy only	Oesophagectomy and gastrectomy
Indication	Malignancy only	Any	Malignancy only
Definition of an involved margin	≤1mm^20^	0 mm^21^	0 mm^21^
Centres enrolled in OGAA	–	8	4

OGAA, Oesophago-Gastric Anastomosis Audit; ECCG, Esophagectomy Complications Consensus Group; DUCA, Dutch Upper Gastrointestinal Cancer Audit.

The OGAA included eight centres that contributed to the ECCG, and four that contributed to the DUCA. Two centres contributed to both the ECCG and DUCA. Of the 137 centres in the OGAA cohort, the approximate case volume was less than 20, 20–60 and over 60 procedures per year in 78, 51, and eight centres, after extrapolating the 9-month numbers collected to provide an annual estimate. Centres in the DUCA and ECCG studies performed an average mean of 37 and 56 oesophagectomies per year respectively.

Comparisons of patient characteristics, tumour staging, treatment, and outcomes between the three cohorts are summarized in *Tables 2–4*, with further detail reported in *Tables S1–S3*.

**Table 2:** Baseline demographics of OGAA, ECCG, and DUCA cohort studies

	% of patients			**Overall *P*** ^*^	***P* for pairwise comparisons** ^§^		
	OGAA	ECCG	DUCA				
					OGAA *versus* ECCG	OGAA *versus* DUCA	ECCG *versus* DUCA
**Male sex**	78.6	77.5	76.0	0.151	–	–	–
**Age (years)**				< 0.001^†^	0.001^¶^	< 0.001^¶^	< 0.001^¶^
< 40	2.5	2.4	0.4				
41–50	8.1	8.0	4.7				
51–60	24.5	26.7	19.5				
61–70	35.8	40.7	45.7				
71-80	26.2	19.7	27.9				
> 80	2.9	2.5	1.8				
**BMI (kg/m^2^)**				0.037^†^	0.039^¶^	1.000^¶^	0.372^¶^
< 18.5	4.2	6.8	2.9				
18.5–25.0	39.9	40.1	40.8				
25.0–30.0	35.3	33.6	39.9				
> 30	20.6	19.5	16.4				
**Smoking status**				–	–	–	–
Never smoked	38.6	–	–				
Ex-smoker (> 6 weeks)	40.3	–	–				
Ex-smoker (< 6 weeks)	5.5	–	–				
Current smoker	15.6	–	–				
**ASA fitness grade**				< 0.001^†^	< 0.001^¶^	< 0.001^¶^	< 0.001^¶^
I	13.3	15.2	15.8				
II	56.1	46.2	62.7				
III	29.7	36.7	21.1				
IV	1.0	1.8	0.4				
V	0.0	0.0	0.0				
**No. of co-morbidities**				< 0.001^‡^	–#	–#	–#
0	59.3	–	46.6				
1	29.5	–	23.8				
≥ 2	11.2	–	29.6				
**ECOG status**				–	–	–	–
0	60.9	–	–				
1	32.8	–	–				
≥ 2	6.3	–	–				
**Diabetes mellitus**				0.089^†^	–	–	–
No	87.9	86.5	85.5				
Uncomplicated	11.2	12.9	13.7				
End-organ damage	0.8	0.6	0.8				
**Myocardial infarction**	6.4	5.4	5.3	0.226	–	–	–
**Congestive heart failure**	3.0	4.6	0.7	< 0.001	0.011	< 0.001	< 0.001
**Chronic pulmonary disease**	13.7	10.5	20.2	< 0.001	0.002	< 0.001	< 0.001
**Peripheral vascular disease**	5.2	6.8	4.5	0.003	0.051	1.000	0.005
**Moderate–severe renal disease**	2.6	1.3	1.3	0.001	0.003	0.016	1.000

Data are reported only as percentages, in order to simplify the table; the associated numerators and denominators are reported in *Table S1*. OGAA, Oesophago-Gastric Anastomosis Audit; ECCG, Esophagectomy Complications Consensus Group; DUCA, Dutch Upper Gastrointestinal Cancer Audit; ECOG, Eastern Cooperative Oncology Group.

* χ^2^ test, except

† Kruskal–Wallis test and

‡ Mann–Whitney *U* test for ordinal variables.

§ Bonferroni-corrected χ^2^ test, except

¶ Bonferroni-corrected Mann–Whitney *U* test for ordinal variables.

# Pairwise comparisons not applicable as data available for only two cohorts.

**Table 3:** Treatment and tumour staging across OGAA, ECCG, and DUCA cohort studies

	% of patients			**Overall** ***P*** ^†^	***P* for pairwise comparisons** ^‡^		
	OGAA	ECCG	DUCA				
					OGAA *versus* ECCG	OGAA *versus* DUCA	ECCG *versus* DUCA
**Neoadjuvant therapy** ^*^				< 0.001	< 0.001	< 0.001	< 0.001
None	24.9	21.1	6.5				
Chemotherapy only	39.1	29.5	5.3				
Radiotherapy only	0.3	0.2	0.4				
CRT	35.6	46.1	87.8				
Definitive CRT	0.0	3.1	0.0				
**Surgical approach**				< 0.001	< 0.001	< 0.001	< 0.001
Open	42.8	52.1	14.2				
MI	57.2	47.9	85.8				
**Open surgery type**				< 0.001	< 0.001	< 0.001	< 0.001
Thoracoabdominal	8.0	0.0	0.0				
Transhiatal	9.7	20.1	47.6				
Transthoracic	82.3	79.9	52.4				
**MI surgery type**				< 0.001	< 0.001	< 0.001	< 0.001
Abdomen and chest	51.6	48.7	79.7				
Abdomen only	42.0	40.2	16.0				
Chest only	6.4	11.1	4.3				
**Anastomosis site**				< 0.001	< 0.001	< 0.001	< 0.001
Chest	77.0	60.7	54.2				
Neck	22.8	37.9	43.0				
Abdomen	0.0	0.0	0.4				
No anastomosis	0.2	1.4	2.4				
**Gastric tube**				< 0.001	< 0.001	0.005	< 0.001
Stomach	100.0	96.0	99.4				
Colon	0.0	1.3	0.3				
Small bowel	0.0	2.7	0.0				
Roux-en-Y	0.0	0.0	0.3				
**Pathological T category** ^*^				< 0.001	0.009	< 0.001	< 0.001
pTx /Tis	2.0	2.1	0.0				
pT0–T2	48.2	53.3	61.2				
pT3	45.8	41.6	37.5				
pT4	4.0	3.0	1.3				
**Pathological N status** ^*^				< 0.001	0.008	< 0.001	0.324
pNx	0.0	0.3	0.3				
pN–	53.8	57.1	60.5				
pN+	46.2	42.6	39.3				
**Pathological M status** ^*^				< 0.001	< 0.001	0.516	< 0.001
pMx	0.9	14.3	1.1				
pM–	96.8	83.9	97.4				
pM+	2.3	1.8	1.5				
**Resection margin** ^*^				–^¶^	–^¶^	–^¶^	0.002^§^
R0	81.8	93.4	95.9				
R1	18.2	6.1	4.1				
R2	0.0	0.5	0.1				

Data are reported only as percentages, in order to simplify the table; the associated numerators and denominators are reported in *Table S2*.

* The Esophagectomy Complications Consensus Group (ECCG) data exclude 119 patients who did not have cancer. OGAA, Oesophago-Gastric Anastomosis Audit; DUCA, Dutch Upper Gastrointestinal Cancer Audit; CRT, chemoradiotherapy MI, minimally invasive.

† χ^2^ test;

‡ Bonferroni-corrected χ^2^ test, except

§ Bonferroni-corrected Mann–Whitney *U* test for ordinal variables.

¶ The OGAA used a different definition of margin involvement from the other cohorts, so comparisons were not meaningful.

**Table 4:** Intraoperative and postoperative outcomes across the OGAA, ECCG, and DUCA cohort studies

	% of patients			**Overall *P*** ^†^	***P* for pairwise comparisons** ^§^		
	OGAA	ECCG	DUCA				
					OGAA *versus* ECCG	OGAA *versus* DUCA	ECCG *versus* DUCA
**Intraoperative complications**	2.5	–	5.5	< 0.001	–^#^	–^#^	–^#^
**Highest Clavien–Dindo grade**				0.752^‡^	–	–	–
No complication	36.4	41.0	37.8				
I	12.0	7.5	9.4				
II	26.2	20.4	23.7				
IIIA	9.7	14.2	12.0				
IIIB	7.1	6.6	8.0				
IVA	4.3	6.4	6.9				
IVB	1.2	1.3	0.7				
V	3.2	2.6	1.7				
**Gastrointestinal complications**	11.5	22.4	24.2	< 0.001	< 0.001	< 0.001	0.501
**Thrombotic complications**	2.9	5.2	2.8	< 0.001	< 0.001	1.000	< 0.001
**Anastomotic leak**				< 0.001^‡^	0.008^¶^	< 0.001^¶^	< 0.001^¶^
No leak	85.8	88.9	81.1				
Type 1	7.0	3.3	5.7				
Type 2	3.4	4.8	8.1				
Type 3	3.8	3.0	5.1				
**Conduit necrosis**				< 0.001^‡^	< 0.001^¶^	< 0.001^¶^	0.702^¶^
No necrosis	97.3	98.8	99.2				
Type 1	1.2	0.1	0.1				
Type 2	0.7	0.3	0.2				
Type 3	0.8	0.9	0.6				
**Combined anastomotic leak/conduit necrosis rate**	14.6	11.4	19.0	< 0.001	0.002	< 0.001	< 0.001
**Chyle leak**	5.4	4.0	4.1	0.043	0.061	0.203	1.000
**Vocal cord injury**	4.6	4.7	7.2	< 0.001	1.000	0.002	0.002
**Respiratory complications**	35.9	27.8	32.7	< 0.001	< 0.001	0.117	0.002
**Cardiac complications**	13.1	16.8	17.1	< 0.001	< 0.001	0.002	1.000
**Diaphragmatic complications**	1.8	2.9	1.9	0.020	0.046	1.000	0.108
**Infective complications**	19.4	14.2	7.4	< 0.001	< 0.001	< 0.001	< 0.001
**Urological complications**	5.9	8.3	4.1	< 0.001	0.003	0.038	< 0.001
**Return to theatre**	12.0	15.7	12.9	< 0.001	< 0.001	1.000	0.033
**30-day readmission** ^*^	11.5	10.2	14.4	< 0.001	0.423	0.022	< 0.001
**30-day mortality**	3.2	2.4	1.7	0.013	0.315	0.011	0.318
**90-day mortality**	4.5	4.5	–	0.967	–^#^	–^#^	–^#^

Data are reported only as percentages, in order to simplify the table; the associated numerators and denominators are reported in *Table S3*.

* The Oesophago-Gastric Anastomosis Audit (OGAA) data exclude 79 patients who either died in hospital, or for whom follow-up was not available. ECCG, Esophagectomy Complications Consensus Group; DUCA, Dutch Upper Gastrointestinal Cancer Audit;

† χ^2^ test, except

‡ Kruskal–Wallis test for ordinal variables.

§ Bonferroni-corrected χ^2^ test, except

¶ Bonferroni-corrected Mann–Whitney *U* test for ordinal variables.

# Pairwise comparisons not applicable as data available for only two cohorts.

### Patient characteristics

Baseline patient characteristics of patients in the three studies are shown in *Table 2*. The three studies had a similar sex distribution, all having a preponderance of men. A significant difference in age was observed, which increased progressively across the ECCG, OGAA, and DUCA cohorts (22.2, 29.1 and 29.7 per cent of the patients respectively were aged over 70 years; *P* < 0.001). The ECCG cohort tended to have lower BMI (BMI at least 25 kg/m^2^: OGAA 55.9 per cent, ECCG 53.1 per cent, DUCA 56.3 per cent; *P* = 0.037), but also had the greatest number of co-morbidities on the basis of ASA grade (ASA grade III or higher: OGAA 30.7 per cent, ECCG 38.5 per cent, DUCA 21.5 per cent; *P* < 0.001).

Specific co-morbidity indices were not reported, although the OGAA and DUCA reported total numbers of co-morbidities, which were significantly higher in the latter (2 or more co-morbidities: OGAA 11.2 per cent, DUCA 29.6 per cent; *P* < 0.001). Assessment of individual co-morbidities showed the DUCA to have the highest rate of chronic pulmonary disease, which was almost twice that of the other cohorts (OGAA 13.7 per cent, ECCG 10.5 per cent, DUCA 20.2 per cent; *P* < 0.001). Of the other co-morbidities reported, the ECCG had the highest rates of both congestive heart failure (OGAA 3.0 per cent, ECCG 4.6 per cent, DUCA 0.7 per cent; *P* < 0.001) and peripheral vascular disease (OGAA 5.2 per cent, ECCG 6.8 per cent, DUCA 4.5 per cent; *P* = 0.03), and the OGAA had the highest rate of moderate-to-severe renal disease (OGAA 2.6 per cent, ECCG 1.3 per cent, DUCA 1.3 per cent; *P* = 0.001). Rates of diabetes mellitus were similar in the three cohorts (OGAA 12.0 per cent, ECCG 13.5 per cent, DUCA 14.5 per cent; *P* = 0.089). Comparison of pathological tumour staging between the three cohorts showed the OGAA to have more advanced tumours, with the highest T and N categories, followed by the ECCG and DUCA (pT3 or higher: OGAA 49.8 per cent, ECCG 44.6 per cent, DUCA 38.8 per cent, *P* < 0.001; pN+: OGAA 46.2 per cent, ECCG 42.6 per cent, DUCA 39.3 per cent; *P* < 0.001) (*Table 3*).

### Treatment variation

Data on treatment and tumour staging across the three cohort studies are presented in *Table 3*. The DUCA contained higher rates of neoadjuvant chemoradiotherapy (CRT) and minimally invasive surgery (CRT rate: OGAA 35.6 per cent, ECCG 46.1 per cent, DUCA 87.8 per cent, *P* < 0.001; minimally invasive approach: OGAA 57.2 per cent, ECCG 47.9 per cent, DUCA 85.8 per cent; *P* < 0.001), with a greater proportion of three-stage operations (abdomen, chest, neck), resulting in higher rates of cervical anastomoses (OGAA 22.8 per cent, ECCG 37.9 per cent, DUCA 43.0 per cent; *P* < 0.001). Comparisons between the OGAA and ECCG showed smaller differences, although the former had higher rates of minimally invasive surgery, whereas the ECCG had higher rates of neoadjuvant CRT and neck anastomoses.

### Postoperative outcomes

**Fig. 1:**
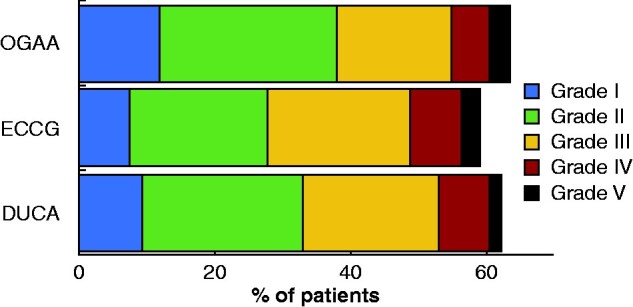
Complication rate by highest Clavien–Dindo grade in OGAA, ACCG, and DUCA studies OGAA, Oesophago-Gastric Anastomosis Audit; ECCG, Esophagectomy Complications Consensus Group; DUCA, Dutch Upper Gastrointestinal Cancer Audit. *P* = 0.752 (Kruskal–Wallis test).

Overall complication rates were similar in the three studies (OGAA 63.6 per cent, ECCG 59.0 per cent, DUCA 62.2 per cent; *P* = 0.752), with no significant difference in complication severity, as classified by the highest Clavien–Dindo grade (*Fig. 1*). Despite this, rates of individual complication types differed between the studies (*Table 4*). For example, the OGAA had the highest rates of respiratory and infective complications, but significantly lower rates of gastrointestinal and cardiac complications. Rates of anastomotic leak and conduit necrosis in the OGAA were 14.2 and 2.7 per cent respectively. Combining these outcomes, it was found that the composite anastomotic leak/conduit necrosis rate differed significantly between the cohorts, being highest in the DUCA, and lowest in the ECCG cohort (OGAA 14.6 per cent, ECCG 11.4 per cent, DUCA 19.0 per cent; *P* < 0.001). There were no significant differences in chyle leak rates between pairs of studies.

Mortality rates at 30 and 90 days were similar in the OGAA and ECCG cohorts (90-day mortality: OGAA 4.5 per cent, ECCG 4.5 per cent; *P* = 0.967). The DUCA did not report 90-day deaths, but had a significantly lower 30-day mortality rate than the other studies (30-day mortality: OGAA 3.2 per cent, ECCG 2.4 per cent, DUCA 1.7 per cent; *P* = 0.013). The Dutch audit had a significantly higher 30-day readmission rate than the other two cohorts (OGAA 11.5 per cent, ECCG 10.2 per cent, DUCA 14.4 per cent; *P* < 0.001).

## Discussion

Postoperative oesophagectomy outcomes across an unselected global cohort have been evaluated and compared with those in previous benchmarking studies from the ECCG and DUCA. Overall rates of complications were comparable in all three studies. The OGAA and ECCG reported similar 30-day mortality rates, but the DUCA had significantly lower mortality rates than the OGAA. This lower mortality rate may reflect a well resourced, centralized, national oesophageal cancer programme facilitating an ability to recognize and rescue the deteriorating patient. Evidence from the Agency for Healthcare Research and Quality Nationwide Readmission Database^23^ showed that failure-to-rescue rates were 21.2 per cent in low-volume centres, compared with 13.4 per cent in high-volume centres.

Patient selection has often been perceived as a key determinant of successful postoperative outcomes. Although definitive CRT has good outcomes for SCC, its efficacy is limited for adenocarcinoma^24–26^. The extent to which patient selection contributed to differences in patient outcome between the three studies remains elusive. The Dutch audit reported the lowest 30-day mortality rate, but contained the highest rate of chronic pulmonary disease and had the greatest proportion of patients aged over 70 years. The OGAA cohort, on the other hand, contained more patients with locally advanced disease (higher T categories and rates of nodal positivity). This might reflect access to, and use of, diagnostic and staging modalities, as well as availability and cultural attitudes to non-surgical treatments (indicated by lower neoadjuvant treatment rates), especially in middle- and low-income societies^13^. The OGAA also had the highest rates of conduit necrosis, which may highlight the challenges of maintaining high surgical standards in lower-volume units. Despite these differences between the three studies, overall complication rates were broadly similar.

There is conflicting evidence regarding the impact of minimally invasive oesophagectomy compared with open surgery on complication rates and other outcomes^27–30^. The TIME and MIRO trials, which demonstrated the superiority of minimally invasive techniques, have further driven rapid adoption^27^^,^^30^^,^^31^, although it should be recognized that transition to a new operative technique can be associated with increased complications that are likely to influence outcomes outside a trial setting^32^^,^^33^. The DUCA involved significantly more minimally invasive surgery than the OGAA and ECCG studies, yet had similar levels of complications. The explanation may be complex. Rates of respiratory complications did not differ significantly between the OGAA and DUCA, despite the significantly higher rates of chronic pulmonary disease in the DUCA cohort, but there were higher rates of open surgery in the OGAA cohort suggesting that the minimally invasive approach may have offset the risk of pulmonary complications in the DUCA cohort.

CRT was also used more frequently in the DUCA cohort, where 87.8 per cent of patients received this treatment. Global variations in neoadjuvant treatment options are largely explained by centres that favour neoadjuvant chemotherapy based on evidence from the MAGIC, OE02, and OE05 trials^34–36^. High uptake of CRT in the Netherlands is likely to have been influenced by the success of the CROSS trial^37^. The absence of increased rates of overall complications in the DUCA cohort compared with the other studies supports existing evidence that neoadjuvant CRT does not increase overall complications^38^.

Anastomotic leakage is generally regarded as a serious complication of oesophagectomy because of the risk of associated sepsis. Leak rates were highest in the DUCA group probably reflecting the high rates of anastomoses performed in the neck, and the highest rates of minimally invasive surgery, both of which are recognized to contribute to higher leak rates^26^^,^^38–41^. The extent to which neoadjuvant CRT contributes to anastomotic leak is controversial^38^^,^^42^^,^^43^, including whether the anastomosis lies within the radiation field or whether the stomach has been irradiated^44^^,^^45^. The combination of chronic pulmonary disease and CRT has been shown to potentially double rates of anastomotic leakage^46^, so these features could also have contributed to the higher leak rates in the DUCA cohort. Despite having the highest rate of anastomotic leak, the DUCA cohort had the lowest 30-day mortality rate, suggesting that anastomotic leaks *per se* are not critical determinants of mortality, or that a cervical leak is less likely to result in death than an intrathoracic leak. The higher readmission rate in the DUCA cohort may indicate a lower threshold for readmission that may itself have influenced outcomes.

A specific focus of the OGAA study was capturing clinically relevant data at a patient-, disease- and operation-specific level to minimize reporting bias in the study. The short duration of data collection was designed to minimize effects due to changes in practice. Despite the overall success in achieving these goals, there are limitations. ECCG and DUCA data were extracted from relevant publications. Data were not available at a patient level; therefore, statistical adjustment for differing preoperative and intraoperative factors was not possible when evaluating outcomes. The inclusion of patients without cancer in the ECCG and DUCA cohorts should be noted, and it was not possible to comment on the success of reported evaluation of neoadjuvant treatments and how this may have influenced short-term outcomes. Different pathological classifications were used to determine margin positivity, precluding comparisons between all three studies^20^^,^^21^^,^^46^. For the OGAA, data were verified by each unit’s lead investigator, although no specific data verification process was undertaken. Previous data verification in national and international observational studies has shown high accuracy^4^^,^^14^^,^^47–49^. A standardized internationally agreed data set covering not only complications, but demographic, oncological, surgical, and pathological data, as developed for pancreatoduodenectomy^50^, seems desirable to make fair comparisons that can result in quality improvements.

The outcome data presented by both the DUCA and ECCG represent high-quality care in centralized and selected settings. The OGAA sought to identify whether these outcomes were achievable in an unselected global cohort. Despite variations in patient demographics, resources, and surgical volumes, the similarity in overall complication rates in all three studies suggests that oesophagectomy can be performed safely at an international level. The present study has also highlighted fundamental shortcomings when comparing international outcome data for oesophagectomy. The development of a standardized data set for future studies should be considered.

## Collaborators

**Writing Committee:** Evans RPT, Kamarajah SK, Nepogodiev D, Bundred J, Hodson J, Blanco-Colino R, Kidane B, Kauppilla J, Wallner B, van Hillegersberg R, Gossage J, Wijnhoven B, Vohra R, Singh P, Griffiths EA

**Data Analysis:** Hodson J, Kamarajah SK, Griffiths EA

**Steering Committee:** Alderson D, Bundred J, Evans RPT, Gossage J, Griffiths EA, Jefferies B, Kamarajah SK, McKay S, Mohamed I, Nepogodiev D, Siaw- Acheampong K, Singh P, van Hillegersberg R, Vohra R, Wanigasooriya K, Whitehouse T.

**National Leads:** Gjata A (Albania), Moreno JI (Argentina), Takeda FR (Brazil), Kidane B (Canada), Guevara Castro R (Colombia), Harustiak T (Czech Republic), Bekele A (Ethiopia), Kechagias A (Finland), Gockel I (Germany), Kennedy A (Ireland), Da Roit A (Italy), Bagajevas A (Lithuania), Azagra JS (Luxembourg), Mahendran HA (Malaysia), Mejía-Fernández L (Mexico), Wijnhoven BPL (Netherlands), El Kafsi J (New Zealand), Sayyed RH (Pakistan), Sousa M (Portugal), Sampaio AS (Portugal), Negoi I (Romania), Blanco R (Spain), Wallner B (Sweden), Schneider PM (Switzerland), Hsu PK (Taiwan), Isik A (Turkey)

**Site Leads:** Gananadha S (The Canberra Hospital, Australia); Wills V (John Hunter Hospital, Australia); Devadas M (Nepean Hospital, Australia); Duong C (Peter MacCallum Cancer Centre, Australia); Talbot M (St George Public and Private Hospitals, Australia); Hii MW (St Vincent's Hospital Melbourne, Australia); Jacobs R (Western Hospital, Victoria, Australia); Andreollo NA (Unicamp University Hospital, Brazil); Johnston B (Saint John Regional Hospital, Canada); Darling G (Toronto General Hospital, University Health Network, Canada); Isaza-Restrepo A (Hospital Universitario Mayor Mederi-Universidad del Rosario, Colombia); Rosero G (Hospital San Ignacio-Universidad Javeriana, Colombia); Arias- Amézquita F (University Hospital Fundacion Santafe de Bogota, Colombia); Raptis D (University Clinic of Erlangen, Germany); Gaedcke J (Medical Unversity Goettingen, Germany); Reim D (Klinikum Rechts der Isar der TU München, Germany); Izbicki J (University Hospital Hamburg Eppendorf, Germany); Egberts JH(University Hospital Kiel, Germany); Dikinis S (Aalborg University Hospital, Denmark); Kjaer DW (Aarhus University Hospital, Denmark); Larsen MH (Odense University Hospital, Denmark); Achiam MP (Copenhagen University hospital Rigshospitalet, Denmark); Saarnio J (Oulu University Hospital, Finland); Theodorou D (Hippokration General Hospital University of Athens, Greece); Liakakos T (Laikon General Hospital, Greece); Korkolis DP (St. Savvas Cancer Hospital, Greece); Robb WB (Beaumont Hospital, Ireland); Collins C (University Hospital Galway, Ireland); Murphy T (Mercy University Hospital, Ireland); Reynolds J (St James's Hospital, Dublin, Ireland); Tonini V (St. Orsola Hospital- University of Bologna, Italy); Migliore M (Polyclinic Hospital University of Catania, Italy); Bonavina L (University of Milano, IRCCS Policlinico San Donato, Department of General and Foregut Surgery, Italy); Valmasoni M (Padova University Hospital-Clinica Chirurgica 3, Italy); Bardini R (Padova University Hospital- General Surgery Department, Italy); Weindelmayer J (Verona Borgo Trento Hospital, Italy); Terashima M (Shizioka Cancer Centre, Japan); White RE (Tenwek Hospital, Kenya); Alghunaim E (Chest Diseases Hospital, Kuwait); Elhadi M (Tripoli, Libya); Leon-Takahashi AM (National Cancer Institute, Mexico); Medina-Franco H (National Institute of Medical Science and Nutrition Salvador Zubirán, Mexico); Lau PC (University Malaya Medical Centre, Malaysia); Okonta KE (Carez Hospital & University of Port-Harcourt Teaching Hospital, Nigeria); Heisterkamp J (Elisabeth-TweeSteden Ziekenhuis Hospital, Netherlands); Rosman C (Radboudumc, Netherlands); van Hillegersberg R (UMC Utrecht, Netherlands); Beban G (Auckland City Hospital, New Zealand); Babor R (Middlemore Hospital, New Zealand); Gordon A (Palmerston North Hospital, New Zealand); Rossaak JI (Tauranga Hospital, Bay of Plenty District Health Board, New Zealand); Pal KMI (Aga Khan University Hospital, Pakistan); Qureshi AU (Services Institute of Medical Sciences, Lahore, Pakistan); Naqi SA (Mayo Hospital, Lahore, Pakistan); Syed AA (Shaukat Khanum Memorial Cancer Hospital & Research Centre Lahore, Pakistan); Barbosa J (Centro Hospitalar São João, Portugal); Vicente CS (Centro Hospitalar Lisboa Central, Portugal); Leite J (Coimbra University Hospital, Portugal); Freire J (Hospital Santa Maria, Portugal); Casaca R (Instituto Português de Oncologia de Lisboa, Portugal); Costa RCT (Instituto Português de Oncologia do Porto, Portugal); Scurtu RR (University Emergency Cluj County Hospital, Romania); Mogoanta SS (Emergency County Hospital of Craiova, Romania); Bolca C (Marius Nasta’ National Institute of Pneumology, Romania); Constantinoiu S (St. Mary Clinical Hospital, Romania); Sekhniaidze D (Tyumen Regional Hospital, Russia); Bjelovic´ M (Department for Minimally Invasive Upper Digestive Surgery, University Hospital for Digestive Surgery, Clinical Center of Serbia, Belgrade, Serbia); So JBY (National University Hospital, Singapore); Gačevski G (University Hospital Maribor, Slovenia); Loureiro C (University Hospital of Basurto (Bilbao), Spain); Pera M (Hospital Universitario del Mar, Spain); Bianchi A (Palma de Mallorca, Spain); Moreno Gijón M (Hospital Universitario Central de Asturias, Spain); Martín Fernández J (Hospital General Universitario De Ciudad Real, Spain); Trugeda Carrera MS (Hospital Universitario Marqués de Valdecilla, Spain); Vallve-Bernal M (Hospital Universitario Nuestra Señora de Candelaria, Spain); Cítores Pascual MA (Hospital Universitario Río Hortega de Valladolid, Spain); Elmahi S (Shaab Teaching Hospital, Sudan), Halldestam I (University Hospital Linköping, Sweden); Hedberg J (Uppsala University Hospital, Sweden); Mönig S (Geneva University Hospital, Switzerland); Gutknecht S (Triemli Hospital Zurich, Switzerland); Tez M (Ankara Numune Hospital, Turkey); Guner A (Karadeniz Technical University, Turkey); Tirnaksiz MB (Hacettepe University Hospital, Turkey); Colak E (University of Health Sciences, Samsun Training and Research Hospital, Turkey); Sevinç B (Usak University Training and Research Hospital, Turkey); Hindmarsh A (Addenbrooke's Hospital, Cambridge, United Kingdom (UK)); Khan I (Aintree University Hospital, Liverpool, UK); Khoo D (Barking Havering and Redbridge NHS Trust, UK); Byrom R (Royal Bournemouth Hospital, UK); Gokhale J (Bradford Royal Infirmary, UK); Wilkerson P (University Hospitals Bristol NHS Foundation Trust, UK); Jain P (Castle Hill Hospital, UK); Chan D (University Hospital of Coventry, UK); Robertson K (University Hospital Crosshouse, UK); Iftikhar S (Royal Derby Hospital, UK); Skipworth R (Edinburgh Royal Infirmary, UK); Forshaw M (Glasgow Royal Infirmary, UK); Higgs S (Gloucester Royal Hospital, UK); Gossage J (Guy's and St Thomas's Hospitals, UK); Nijjar R (Heartlands Hospital, UK); Viswanath YKS (James Cook University Hospital, UK); Turner P (Lancashire Teaching Hospitals NHS Foundation Trust, UK); Dexter S (Leeds Teaching Hospitals NHS Trust, UK); Boddy A (University Hospitals of Leicester NHS Trust, UK); Allum WH (Royal Marsden Hospital, UK); Oglesby S (Ninewells Hospital, UK); Cheong E (Norfolk and Norwich University Hospital, UK); Beardsmore D (University Hospital of North Midlands, UK); Vohra R (Nottingham University Hospital, UK); Maynard N (Oxford University Hospitals, UK); Berrisford R (Plymouth Hospitals NHS Trust, UK); Mercer S (Queen Alexandra Hospital, Portsmouth, UK); Puig S (Queen Elizabeth Hospital Birmingham, UK); Melhado R (Salford Royal Foundation Trust, UK); Kelty C (Sheffield Teaching Hospitals NHS Foundation Trust, UK); Underwood T (University Hospital Southampton NHS Foundation Trust, UK); Dawas K (University College Hospital, UK); Lewis W (University Hospital of Wales, UK); Al-Bahrani A (Watford General Hospital); Bryce G (University Hospital Wishaw, UK); Thomas M (Mayo Clinic in Florida, United States of America (USA)); Arndt AT (Rush University Medical Center, USA); Palazzo F (Thomas Jefferson University, USA); Meguid RA (University of Colorado Hospital, USA)

**Oesophago-Gastric Anastomosis Study Group:** Fergusson J, Beenen E, Mosse C, Salim J (The Canberra Hospital, Australia); Cheah S, Wright T, Cerdeira MP, McQuillan P (John Hunter Hospital, Australia); Richardson M, Liem H (Nepean Hospital, Australia); Spillane J, Yacob M, Albadawi F, Thorpe T, Dingle A, Cabalag C (Peter MacCallum Cancer Centre, Australia); Loi K, Fisher OM (St George Public and Private Hospitals, Australia); Ward S, Read M, Johnson M (St Vincent's Hospital Melbourne, Australia); Bassari R, Bui H (Western Hospital, Victoria); Cecconello I, Sallum RAA, da Rocha JRM (Hospital das Clinicas, University of Sao Paulo School of Medicine, Brazil); Lopes LR, Tercioti V Jr, Coelho JDS, Ferrer JAP (Unicamp University Hospital, Brazil); Buduhan G, Tan L, Srinathan S (Health Sciences Centre (Winnipeg)); Shea P (Saint John Regional Hospital, Canada); Yeung J, Allison F, Carroll P (Toronto General Hospital, University Health Network, Canada); Vargas-Barato F, Gonzalez F, Ortega J, Nino-Torres L, Beltrán-García TC (Hospital Universitario Mayor Mederi-Universidad del Rosario, Colombia); Castilla L, Pineda M (Hospital San Ignacio-Universidad Javeriana, Colombia); Bastidas A, Gómez-Mayorga J, Cortés N, Cetares C, Caceres S, Duarte S (University Hospital Fundacion Santafe de Bogota, Colombia); Pazdro A, Snajdauf M, Faltova H, Sevcikova M (Motol University Hospital, Prague, Czech Republic); Mortensen PB (Aalborg University Hospital, Denmark); Katballe N, Ingemann T, Morten B, Kruhlikava I (Aarhus University Hospital, Denmark); Ainswort AP, Stilling NM, Eckardt J (Odense University Hospital, Denmark); Holm J, Thorsteinsson M, Siemsen M, Brandt B (Copenhagen University hospital Rigshospitalet, Denmark); Nega B, Teferra E, Tizazu A (Tikur Anbessa Specialized Hospital, Ethiopa); Kauppila JS, Koivukangas V, Meriläinen S (Oulu University Hospital, Finland); Gruetzmann R, Krautz C, Weber G, Golcher H (University Clinic of Erlangen, Germany); Emons G, Azizian A, Ebeling M (Medical University Goettingen, Germany); Niebisch S, Kreuser N, Albanese G, Hesse J (Universitätklinium Leipzig, Germany); Volovnik L, Boecher U (Klinikum Rechts der Isar der TU München, Germany); Reeh M (University Hospital Hamburg Eppendorf, Germany); Triantafyllou S (Hippokration General Hospital University of Athens, Greece); Schizas D, Michalinos A, Mpali E, Mpoura M, Charalabopoulos A (Laikon General Hospital, Greece); Manatakis DK, Balalis D (St. Savvas Cancer Hospital, Greece); Bolger J, Baban C, Mastrosimone A (Beaumont Hospital, Ireland); McAnena O, Quinn A (University Hospital Galway, Ireland); Ó Súilleabháin CB, Hennessy MM, Ivanovski I, Khizer H (Mercy University Hospital, Ireland); Ravi N, Donlon N (St James's Hospital, Dublin, Ireland); Cervellera M, Vaccari S, Bianchini S, Sartarelli l (St. Orsola Hospital- University of Bologna, Italy); Asti E, Bernardi D (University of Milano, IRCCS Policlinico San Donato, Department of General and Foregut Surgery, Italy); Merigliano S, Provenzano L (Padova University Hospital - Clinica Chirurgica, Italy); Scarpa M, Saadeh L, Salmaso B (Padova University Hospital- General Surgery Department, Italy); De Manzoni G, Giacopuzzi S, La Mendola R, De Pasqual CA (Verona Borgo Trento Hospital, Italy); Tsubosa Y, Niihara M, Irino T, Makuuchi R, Ishii K (Shizioka Cancer Centre, Japan); Mwachiro M, Fekadu A, Odera A, Mwachiro E (Tenwek Hospital, Kenya); AlShehab D (Chest diseases hospital, Kuwait); Ahmed HA, Shebani AO, Elhadi A, Elnagar FA, Elnagar HF (Tripoli, Libya); Makkai-Popa ST (Centre Hospitalier de Luxembourg, Luxembourg); Wong LF (University Malaya Medical Centre, Malaysia); Yunrong T, Thanninalai S, Aik HC, Soon PW, Huei TJ (Hospital Sultanah Aminah, Malaysia); Basave HNL (National Cancer Institute, Mexico); Cortés-González R (Instituto Nacional de Ciencias Médicas y Nutrición ’Salvador Zubirán’, Mexico); Lagarde SM, van Lanschot JJB, Cords C (Erasmus University Medical Center, Rotterdam, Netherlands); Jansen WA, Martijnse I, Matthijsen R (Elisabeth-TweeSteden Ziekenhuis Hospital, Netherlands); Bouwense S, Klarenbeek B, Verstegen M, van Workum F (Radboudumc, Netherlands); Ruurda JP, van der Sluis PC, de Maat M (UMC Utrecht, Netherlands); Evenett N, Johnston P, Patel R (Auckland City Hospital, New Zealand); MacCormick A (Middlemore Hospital, New Zealand); Young M (Palmerston North Hospital); Smith B (Tauranga Hospital, Bay of Plenty District Health Board, New Zealand); Ekwunife C (Carez Hospital & University of Port-Harcourt Teaching Hospital, Nigeria); Memon AH, Shaikh K, Wajid A (Aga Khan University Hospital, Pakistan); Khalil N, Haris M, Mirza ZU, Qudus SBA (Services Institute of Medical Sciences, Lahore, Pakistan); Sarwar MZ, Shehzadi A, Raza A, Jhanzaib MH (Mayo Hospital, Lahore, Pakistan); Farmanali J, Zakir Z (Patel Hospital, Pakistan); Shakeel O, Nasir I, Khattak S, Baig M, Noor MA, Ahmed HH, Naeem A (Shaukat Khanum Memorial Cancer Hospital & Research Centre Lahore, Pakistan); Pinho AC, da Silva R (Centro Hospitalar Lisboa Central, Portugal), Bernardes A, Campos JC (Coimbra University Hospital, Portugal); Matos H, Braga T (Hospital Santa Maria, Portugal); Monteiro C, Ramos P, Cabral F (Instituto Português de Oncologia de Lisboa, Portugal); Gomes MP, Martins PC, Correia AM, Videira JF (Instituto Português de Oncologia do Porto, Portugal); Ciuce C, Drasovean R, Apostu R, Ciuce C (University Emergency Cluj County Hospital, Romania); Paitici S, Racu AE, Obleaga CV (Emergency County Hospital of Craiova, Romania); Beuran M, Stoica B, Ciubotaru C, Negoita V (Emergency Hospital of Bucharest, Romania); Cordos I (Marius Nasta’ National Institute of Pneumology, Romania); Birla RD, Predescu D, Hoara PA, Tomsa R (St. Mary Clinical Hospital, Romania); Shneider V, Agasiev M, Ganjara I (Tyumen Regional Hospital, Russia); Gunjic´ D, Veselinovic´ M, Babič T (Department for Minimally Invasive Upper Digestive Surgery, University Hospital for Digestive Surgery, Clinical Center of Serbia, Belgrade, Serbia); Chin TS, Shabbir A, Kim G (National University Hospital, Singapore); Crnjac A, Samo H (University Hospital Maribor, Slovenia); Díez del Val I, Leturio S (University Hospital of Basurto (Bilbao), Spain); Díez del Val I, Leturio S, Ramón JM, Dal Cero M, Rifá S, Rico M (Hospital Universitario del Mar, Spain); Pagan Pomar A, Martinez Corcoles JA (Palma de Mallorca, Spain); Rodicio Miravalles JL, Pais SA, Turienzo SA, Alvarez LS (Hospital Universitario Central de Asturias, Spain); Campos PV, Rendo AG, García SS, Santos EPG (Hospital General Universitario De Ciudad Real, Spain); Martínez ET, Fernández Díaz MJ, Magadán Álvarez C (Hospital Universitario Marqués de Valdecilla, Spain); Concepción Martín V, Díaz López C, Rosat Rodrigo A, Pérez Sánchez LE (Hospital Universitario Nuestra Señora de Candelaria, Spain); Bailón Cuadrado M, Tinoco Carrasco C, Choolani Bhojwani E, Sánchez DP (Hospital Universitario Río Hortega de Valladolid, Spain); Ahmed ME (Shaab Teaching Hospital, Sudan); Dzhendov T (University Hospital Linköping, Sweden); Lindberg F, Rutegård M (Umeå University Hospital, Sweden); Sundbom M (Uppsala University Hospital, Sweden); Mickael C, Colucci N (Geneva University Hospital, Switzerland); Schnider A (Triemli Hospital Zurich, Switzerland); Er S (Ankara Numune Hospital, Turkey); Kurnaz E (Erzincan University Hospital, Turkey); Turkyilmaz S, Turkyilmaz A, Yildirim R, Baki BE (Karadeniz Technical University, Turkey); Akkapulu N (Hacettepe University Hospital, Turkey); Karahan O, Damburaci N (Usak University Training and Research Hospital, Turkey); Hardwick R, Safranek P, Sujendran V, Bennett J, Afzal Z (Addenbrooke's Hospital, Cambridge, United Kingdom (UK)); Shrotri M, Chan B, Exarchou K, Gilbert T (Aintree University Hospital, Liverpool, UK); Amalesh T, Mukherjee D, Mukherjee S, Wiggins TH (Barking Havering and Redbridge NHS Trust, UK); Kennedy R, McCain S, Harris A, Dobson G (Belfast City Hospital, UK); Davies N, Wilson I, Mayo D, Bennett D (Royal Bournemouth Hospital, UK); Young R, Manby P (Bradford Royal Infirmary, UK); Blencowe N, Schiller M, Byrne B (University Hospitals Bristol NHS Foundation Trust, UK); Mitton D, Wong V, Elshaer A, Cowen M (Castle Hill Hospital, UK); Menon V, Tan LC, McLaughlin E, Koshy R (University Hospital of Coventry, UK); Sharp C (University Hospital Crosshouse, UK); Brewer H, Das N, Cox M, Al Khyatt W, Worku D (Royal Derby Hospital, UK); Iqbal R, Walls L, McGregor R (Edinburgh Royal Infirmary, UK); Fullarton G, Macdonald A, MacKay C, Craig C (Glasgow Royal Infirmary, UK); Dwerryhouse S, Hornby S, Jaunoo S, Wadley M (Gloucester Royal Hospital, UK); Baker C, Saad M, Kelly M, Davies A, Di Maggio F (Guy's and St Thomas's Hospitals, UK); McKay S, Mistry P, Singhal R, Tucker O, Kapoulas S, Powell-Brett S (Heartlands Hospital, UK); Davis P, Bromley G, Watson L (James Cook University Hospital, UK); Verma R, Ward J, Shetty V, Ball C, Pursnani K (Lancashire Teaching Hospitals NHS Foundation Trust, UK); Sarela A, Sue Ling H, Mehta S, Hayden J, To N (Leeds Teaching Hospitals NHS Trust, UK); Palser T, Hunter D, Supramaniam K, Butt Z, Ahmed A (University Hospitals of Leicester NHS Trust, UK); Kumar S, Chaudry A, Moussa O (Royal Marsden Hospital, UK); Kordzadeh A, Lorenzi B (Mid and South Essex NHS Foundation Trust, UK) Wilson M, Patil P, Noaman I (Ninewells Hospital, UK); Willem J (Norfolk and Norwich University Hospital); Bouras G, Evans R, Singh M, Warrilow H, Ahmad A (University Hospital of North Midlands, UK); Tewari N, Yanni F, Couch J, Theophilidou E, Reilly JJ, Singh P (Nottingham University Hospital, UK); van Boxel Gijs, Akbari K, Zanotti D, Sgromo B (Oxford University Hospitals); Sanders G, Wheatley T, Ariyarathenam A, Reece-Smith A, Humphreys L (Plymouth Hospitals NHS Trust, UK); Choh C, Carter N, Knight B, Pucher P (Queen Alexandra Hospital, Portsmouth, UK); Athanasiou A, Mohamed I, Tan B, Abdulrahman M (Queen Elizabeth Hospital Birmingham, UK); Vickers J, Akhtar K, Chaparala R, Brown R, Alasmar MMA (Salford Royal Foundation Trust, UK); Ackroyd R, Patel K, Tamhankar A, Wyman A (Sheffield Teaching Hospitals NHS Foundation Trust, UK); Walker R, Grace B (University Hospital Southampton NHS Foundation Trust, UK); Abbassi N, Slim N, Ioannidi L (University College Hospital, UK); Blackshaw G, Havard T, Escofet X, Powell A (University Hospital of Wales, UK); Owera A, Rashid F, Jambulingam P, Padickakudi J (Watford General Hospital, UK); Ben-Younes H, Mccormack K (University Hospital Wishaw, UK); Makey IA (Mayo Clinic in Florida, United States of America (USA)); Karush MK, Seder CW, Liptay MJ, Chmielewski G (Rush University Medical Center, USA); Rosato EL, Berger AC, Zheng R, Okolo E (Thomas Jefferson University, USA); Singh A, Scott CD, Weyant MJ, Mitchell JD (University of Colorado Hospital, USA).

## Supplementary Material

zrab010_Supplementary_Data
