# Erratum to: Comparison of short-term outcomes from the International Oesophago-Gastric Anastomosis Audit (OGAA), the Esophagectomy Complications Consensus Group (ECCG), and the Dutch Upper Gastrointestinal Cancer Audit (DUCA)

**DOI:** 10.1093/bjsopen/zrac027

**Published:** 2022-02-18

**Authors:** Oesophago-Gastric Anastomosis Study Group

BJS Open, 2022, https://doi.org/10.1093/bjsopen/zrab010

In the originally published version of this manuscript, group author contributing authors were omitted from the manuscript in error. This error has been corrected.

