# Supplementary material for: Comparison of short-term outcomes from the International Oesophago-Gastric Anastomosis Audit (OGAA), the Esophagectomy Complications Consensus Group (ECCG), and the Dutch Upper Gastrointestinal Cancer Audit (DUCA)
Source: BJS Open. 2021 May 22;5(3):zrab010. doi: 10.1093/bjsopen/zrab010 (PMC8140199; doi:10.1093/bjsopen/zrab010)
Supplement: zrab010_Supplementary_Data [file zrab010_supplementary_data.docx]

**Table S1 – Baseline Demographics of the OGAA, ECCG, and DUCA Cohort Studies**

|  | **OGAA** | **ECCG** | **DUCA** | **Overall p-Value** | **Pairwise p-Values** | | |
| --- | --- | --- | --- | --- | --- | --- | --- |
|  |  |  |  |  | ***OGAA vs. ECCG*** | ***OGAA vs. DUCA*** | ***ECCG vs. DUCA*** |
| Gender (% Male) | 78.6% (1767/2247) | 77.5% (2096/2703) | 76.0% (1228/1616) | 0.151 | - | - | - |
| Age (Years) |  |  |  | **<0.001*** | **0.001*** | **<0.001*** | **<0.001*** |
| *<40* | 2.5% (57/2247) | 2.4% (66/2703) | 0.4% (6/1617) |  |  |  |  |
| *41-50* | 8.1% (182/2247) | 8.0% (217/2703) | 4.7% (76/1617) |  |  |  |  |
| *51-60* | 24.5% (550/2247) | 26.7% (721/2703) | 19.5% (316/1617) |  |  |  |  |
| *61-70* | 35.8% (804/2247) | 40.7% (1100/2703) | 45.7% (739/1617) |  |  |  |  |
| *71-80* | 26.2% (589/2247) | 19.7% (532/2703) | 27.9% (451/1617) |  |  |  |  |
| *>80* | 2.9% (65/2247) | 2.5% (67/2703) | 1.8% (29/1617) |  |  |  |  |
| Body Mass Index (kg/m^2^) |  |  |  | **0.037*** | **0.039*** | 1.000* | 0.372* |
| *<18.5* | 4.2% (95/2240) | 6.8% (184/2703) | 2.9% (47/1611) |  |  |  |  |
| *18.5 - 25.0* | 39.9% (894/2240) | 40.1% (1085/2703) | 40.8% (657/1611) |  |  |  |  |
| *25.0 - 30.0* | 35.3% (790/2240) | 33.6% (908/2703) | 39.9% (642/1611) |  |  |  |  |
| *>30* | 20.6% (461/2240) | 19.5% (526/2703) | 16.4% (265/1611) |  |  |  |  |
| Smoking Status |  |  |  | *NA* | *-* | *-* | *-* |
| *Never* | 38.6% (842/2183) | - | - |  |  |  |  |
| *Ex-Smoker (>6 weeks)* | 40.3% (880/2183) | - | - |  |  |  |  |
| *Ex-Smoker (<6 weeks)* | 5.5% (121/2183) | - | - |  |  |  |  |
| *Current* | 15.6% (340/2183) | - | - |  |  |  |  |
| ASA Grade |  |  |  | **<0.001*** | **<0.001*** | **<0.001*** | **<0.001*** |
| *Grade 1* | 13.3% (298/2246) | 15.2% (412/2703) | 15.8% (255/1614) |  |  |  |  |
| *Grade 2* | 56.1% (1260/2246) | 46.2% (1249/2703) | 62.7% (1012/1614) |  |  |  |  |
| *Grade 3* | 29.7% (666/2246) | 36.7% (992/2703) | 21.1% (340/1614) |  |  |  |  |
| *Grade 4* | 1.0% (22/2246) | 1.8% (49/2703) | 0.4% (7/1614) |  |  |  |  |
| *Grade 5* | 0.0% (0/2246) | 0.0% (1/2703) | 0.0% (0/1614) |  |  |  |  |
| Number of Comorbidities |  |  |  | **<0.001*** | *NA* | *NA* | *NA* |
| *0* | 59.3% (1333/2247) | - | 46.6% (754/1617) |  |  |  |  |
| *1* | 29.5% (662/2247) | - | 23.8% (385/1617) |  |  |  |  |
| *2+* | 11.2% (252/2247) | - | 29.6% (478/1617) |  |  |  |  |
| ECOG Status |  |  |  | *NA* | *-* | *-* | *-* |
| *0* | 60.9% (1364/2241) | - | - |  |  |  |  |
| *1* | 32.8% (735/2241) | - | - |  |  |  |  |
| *2+* | 6.3% (142/2241) | - | - |  |  |  |  |
| Diabetes Mellitus |  |  |  | 0.089* | *-* | *-* | *-* |
| *No* | 87.9% (1976/2247) | 86.5% (2340/2704) | 85.5% (1383/1617) |  |  |  |  |
| *Uncomplicated* | 11.2% (252/2247) | 12.9% (348/2704) | 13.7% (221/1617) |  |  |  |  |
| *End Organ Damage* | 0.8% (19/2247) | 0.6% (16/2704) | 0.8% (13/1617) |  |  |  |  |
| Myocardial Infarction | 6.4% (144/2247) | 5.4% (146/2704) | 5.3% (86/1617) | 0.226 | *-* | *-* | *-* |
| Congestive Heart Failure | 3.0% (67/2247) | 4.6% (124/2704) | 0.7% (12/1617) | **<0.001** | **0.011** | **<0.001** | **<0.001** |
| Chronic Pulmonary Disease | 13.7% (307/2247) | 10.5% (285/2704) | 20.2% (326/1617) | **<0.001** | **0.002** | **<0.001** | **<0.001** |
| Peripheral Vascular Disease | 5.2% (117/2247) | 6.8% (185/2704) | 4.5% (73/1617) | **0.003** | 0.051 | 1.000 | **0.005** |
| Moderate to Severe Renal Disease | 2.6% (58/2247) | 1.3% (35/2704) | 1.3% (21/1617) | **0.001** | **0.003** | **0.016** | 1.000 |

*Overall p-Values are from Chi-square tests, with pairwise comparisons from Bonferroni-corrected Chi-square tests unless stated otherwise. Bold p-values are significant at p<0.05. NA=pairwise comparisons are not applicable, as data are only available for two cohorts. *p-Values from Kruskal-Wallis tests (Overall) or Bonferroni-corrected Mann-Whitney tests (Pairwise), as the factor is ordinal. ASA – American Society of Anaesthesiologists, ECOG – Eastern Cooperative Oncology Group.*

**Table S2 – Treatment and Tumour Staging across the OGAA, ECCG, and DUCA cohort studies**

|  | **OGAA** | **ECCG** | **DUCA** | **Overall p-Value** | **Pairwise p-Values** | | |
| --- | --- | --- | --- | --- | --- | --- | --- |
|  |  |  |  |  | ***OGAA vs. ECCG*** | ***OGAA vs. DUCA*** | ***ECCG vs. DUCA*** |
| Neoadjuvant therapy** |  |  |  | **<0.001** | **<0.001** | **<0.001** | **<0.001** |
| *None* | 24.9% (560/2247) | 21.1% (545/2585)** | 6.5% (105/1614) |  |  |  |  |
| *Chemo Only* | 39.1% (879/2247) | 29.5% (763/2585)** | 5.3% (86/1614) |  |  |  |  |
| *RT Only* | 0.3% (7/2247) | 0.2% (5/2585)** | 0.4% (6/1614) |  |  |  |  |
| *CRT* | 35.6% (801/2247) | 46.1% (1192/2585)** | 87.8% (1417/1614) |  |  |  |  |
| *Definitive CRT* | 0.0% (0/2247) | 3.1% (80/2585)** | 0.0% (0/1614) |  |  |  |  |
| Surgical Approach |  |  |  | **<0.001** | **<0.001** | **<0.001** | **<0.001** |
| *Open* | 42.8% (954/2229) | 52.1% (1407/2703) | 14.2% (229/1617) |  |  |  |  |
| *MI* | 57.2% (1275/2229) | 47.9% (1296/2703) | 85.8% (1388/1617) |  |  |  |  |
| Surgery Type (Open Only) |  |  |  | **<0.001** | **<0.001** | **<0.001** | **<0.001** |
| *Thoracoabdominal* | 8.0% (76/954) | 0.0% (0/1407) | 0.0% (0/229) |  |  |  |  |
| *Transhiatal* | 9.7% (93/954) | 20.1% (283/1407) | 47.6% (109/229) |  |  |  |  |
| *Transthoracic* | 82.3% (785/954) | 79.9% (1124/1407) | 52.4% (120/229) |  |  |  |  |
| Surgery Type (MI Only) |  |  |  | **<0.001** | **<0.001** | **<0.001** | **<0.001** |
| *Abdomen and Chest* | 51.6% (658/1275) | 48.7% (631/1296) | 79.7% (1106/1388) |  |  |  |  |
| *Abdomen Only* | 42.0% (536/1275) | 40.2% (521/1296) | 16.0% (222/1388) |  |  |  |  |
| *Chest Only* | 6.4% (81/1275) | 11.1% (144/1296) | 4.3% (60/1388) |  |  |  |  |
| Anastomosis Site |  |  |  | **<0.001** | **<0.001** | **<0.001** | **<0.001** |
| *Chest* | 77.0% (1726/2242) | 60.7% (1641/2703) | 54.2% (876/1617) |  |  |  |  |
| *Neck* | 22.8% (512/2242) | 37.9% (1025/2703) | 43.0% (696/1617) |  |  |  |  |
| *Abdomen* | 0.0% (0/2242) | 0.0% (0/2703) | 0.4% (7/1617) |  |  |  |  |
| *Not Performed* | 0.2% (4/2242) | 1.4% (37/2703) | 2.4% (38/1617) |  |  |  |  |
| Gastric Tube |  |  |  | **<0.001** | **<0.001** | **0.005** | **<0.001** |
| *Stomach* | 100.0% (2241/2241) | 96.0% (2564/2670) | 99.4% (1567/1576) |  |  |  |  |
| *Colon* | 0.0% (0/2241) | 1.3% (34/2670) | 0.3% (4/1576) |  |  |  |  |
| *Small Bowel* | 0.0% (0/2241) | 2.7% (72/2670) | 0.0% (0/1576) |  |  |  |  |
| *Roux-en-Y* | 0.0% (0/2241) | 0.0% (0/2670) | 0.3% (5/1576) |  |  |  |  |
| T-Stage (On Pathology)** |  |  |  | **<0.001** | **0.009** | **<0.001** | **<0.001** |
| *Tx / Tis* | 2.0% (45/2238) | 2.1% (54/2582)** | 0.0% (0/1579) |  |  |  |  |
| *T0 - T2* | 48.2% (1079/2238) | 53.3% (1375/2582)** | 61.2% (966/1579) |  |  |  |  |
| *T3* | 45.8% (1025/2238) | 41.6% (1075/2582)** | 37.5% (592/1579) |  |  |  |  |
| *T4* | 4.0% (89/2238) | 3.0% (78/2582)** | 1.3% (21/1579) |  |  |  |  |
| N-Stage (On Pathology)** |  |  |  | **<0.001** | **0.008** | **<0.001** | 0.324 |
| *Nx* | 0.0% (0/2247) | 0.3% (7/2585)** | 0.3% (4/1583) |  |  |  |  |
| *N-* | 53.8% (1210/2247) | 57.1% (1477/2585)** | 60.5% (957/1583) |  |  |  |  |
| *N+* | 46.2% (1037/2247) | 42.6% (1101/2585)** | 39.3% (622/1583) |  |  |  |  |
| M-Stage (On Pathology)** |  |  |  | **<0.001** | **<0.001** | 0.516 | **<0.001** |
| *Mx* | 0.9% (21/2247) | 14.3% (369/2585)** | 1.1% (18/1569) |  |  |  |  |
| *M-* | 96.8% (2175/2247) | 83.9% (2170/2585)** | 97.4% (1528/1569) |  |  |  |  |
| *M+* | 2.3% (51/2247) | 1.8% (46/2585)** | 1.5% (23/1569) |  |  |  |  |
| Resection Margin** |  |  |  | *NA**** | *NA**** | *NA**** | **0.002*** |
| *R0* | 81.8% (1839/2247) | 93.4% (2414/2585)** | 95.9% (1532/1598) |  |  |  |  |
| *R1* | 18.2% (408/2247) | 6.1% (157/2585)** | 4.1% (65/1598) |  |  |  |  |
| *R2* | 0.0% (0/2247) | 0.5% (14/2585)** | 0.1% (1/1598) |  |  |  |  |

*Overall p-Values are from Chi-square tests, with pairwise comparisons from Bonferroni-corrected Chi-square tests unless stated otherwise. Bold p-values are significant at p<0.05. NA=pairwise comparisons are not applicable, as data are only available for two cohorts. MI=Minimally Invasive. *p-Values from Kruskal-Wallis tests (Overall) or Bonferroni-corrected Mann-Whitney tests (Pairwise), as the factor is ordinal **The ECCG data excludes N=119 patients who did not have cancer. ***OGAA used a different definition of margin involvement to the other cohorts; hence comparisons were not meaningful. RT – Radiotherapy, CRT - Chemoradiotherapy.*

**Table S3 – Intra-Operative and Post-Operative Outcomes across the OGAA, ECCG, and DUCA cohort studies**

|  | **OGAA** | **ECCG** | **DUCA** |  | **Pairwise p-Values** | | |
| --- | --- | --- | --- | --- | --- | --- | --- |
|  |  |  |  | **Overall p-Value** | ***OGAA vs. ECCG*** | ***OGAA***  ***vs.***  ***DUCA*** | ***ECCG vs. DUCA*** |
| Intra-Operative Complications | 2.5% (57/2247) | - | 5.5% (89/1617) | **<0.001** | *NA* | *NA* | *NA* |
| Highest Clavien-Dindo Grade |  |  |  | 0.752* | *-* | *-* | *-* |
| *None* | 36.4% (818/2247) | 41.0% (1109/2704) | 37.8% (605/1602) |  |  |  |  |
| *Grade I* | 12.0% (269/2247) | 7.5% (204/2704) | 9.4% (150/1602) |  |  |  |  |
| *Grade II* | 26.2% (588/2247) | 20.4% (551/2704) | 23.7% (379/1602) |  |  |  |  |
| *Grade IIIA* | 9.7% (217/2247) | 14.2% (385/2704) | 12.0% (192/1602) |  |  |  |  |
| *Grade IIIB* | 7.1% (159/2247) | 6.6% (178/2704) | 8.0% (128/1602) |  |  |  |  |
| *Grade IVA* | 4.3% (97/2247) | 6.4% (173/2704) | 6.9% (110/1602) |  |  |  |  |
| *Grade IVB* | 1.2% (28/2247) | 1.3% (35/2704) | 0.7% (11/1602) |  |  |  |  |
| *Grade V* | 3.2% (71/2247) | 2.6% (69/2704) | 1.7% (27/1602) |  |  |  |  |
| Gastrointestinal Complications | 11.5% (258/2247) | 22.4% (606/2704) | 24.2% (392/1617) | **<0.001** | **<0.001** | **<0.001** | 0.501 |
| Thrombotic Complications | 2.9% (65/2247) | 5.2% (141/2704) | 2.8% (45/1617) | **<0.001** | **<0.001** | 1.000 | **<0.001** |
| Anastomotic Leak |  |  |  | **<0.001*** | **0.008*** | **<0.001*** | **<0.001*** |
| *No leak* | 85.8% (1928/2247) | 88.9% (2403/2704) | 81.1% (1310/1616) |  |  |  |  |
| *Type 1 leak* | 7.0% (158/2247) | 3.3% (90/2704) | 5.7% (92/1616) |  |  |  |  |
| *Type 2 leak* | 3.4% (76/2247) | 4.8% (131/2704) | 8.1% (131/1616) |  |  |  |  |
| *Type 3 leak* | 3.8% (85/2247) | 3.0% (80/2704) | 5.1% (83/1616) |  |  |  |  |
| Conduit Necrosis |  |  |  | **<0.001*** | **<0.001*** | **<0.001*** | 0.702* |
| *No necrosis* | 97.3% (2186/2246) | 98.8% (2672/2704) | 99.2% (1604/1617) |  |  |  |  |
| *Type 1 necrosis* | 1.2% (28/2246) | 0.1% (2/2704) | 0.1% (1/1617) |  |  |  |  |
| *Type 2 necrosis* | 0.7% (15/2246) | 0.3% (7/2704) | 0.2% (3/1617) |  |  |  |  |
| *Type 3 necrosis* | 0.8% (17/2246) | 0.9% (23/2704) | 0.6% (9/1617) |  |  |  |  |
| Combined AL/CN Rate | 14.6% (329/2247) | 11.4% (307/2704) | 19.0% (307/1617) | **<0.001** | **0.002** | **<0.001** | **<0.001** |
| Chyle Leak |  |  |  | **0.023*** | **0.036*** | 0.129* | 1.000* |
| *None* | 94.6% (2125/2247) | 96.0% (2595/2704) | 95.9% (1550/1617) |  |  |  |  |
| *1A* | 1.5% (34/2247) | 3.0% (81/2704) | 3.4% (55/1617) |  |  |  |  |
| *1B* | 0.4% (8/2247) | 0.2% (6/2704) | 0.4% (6/1617) |  |  |  |  |
| *2A* | 0.8% (17/2247) | 0.4% (12/2704) | 0.2% (4/1617) |  |  |  |  |
| *2B* | 0.4% (9/2247) | 0.1% (4/2704) | 0.1% (1/1617) |  |  |  |  |
| *3A* | 0.7% (16/2247) | 0.1% (2/2704) | 0.0% (0/1617) |  |  |  |  |
| *3B* | 1.7% (38/2247) | 0.1% (4/2704) | 0.1% (1/1617) |  |  |  |  |
| Vocal Cord Injury |  |  |  | **<0.001*** | 1.000* | **0.001*** | **0.002*** |
| *None* | 95.4% (2144/2247) | 95.3% (2578/2704) | 92.8% (1478/1592) |  |  |  |  |
| *1A* | 3.4% (76/2247) | 2.5% (67/2704) | 4.3% (68/1592) |  |  |  |  |
| *1B* | 0.5% (11/2247) | 0.4% (10/2704) | 0.2% (3/1592) |  |  |  |  |
| *2A* | 0.5% (12/2247) | 0.4% (11/2704) | 0.9% (14/1592) |  |  |  |  |
| *2B* | 0.0% (1/2247) | 0.2% (6/2704) | 0.5% (8/1592) |  |  |  |  |
| *3A* | 0.0% (0/2247) | 0.4% (12/2704) | 0.1% (2/1592) |  |  |  |  |
| *3B* | 0.1% (3/2247) | 0.7% (20/2704) | 1.2% (19/1592) |  |  |  |  |
| Respiratory Complications | 35.9% (807/2247) | 27.8% (752/2704) | 32.7% (529/1617) | **<0.001** | **<0.001** | 0.117 | **0.002** |
| Cardiac Complications | 13.1% (294/2247) | 16.8% (455/2704) | 17.1% (276/1617) | **<0.001** | **<0.001** | **0.002** | 1.000 |
| Diaphragmatic Complications | 1.8% (41/2247) | 2.9% (78/2704) | 1.9% (30/1617) | **0.020** | **0.046** | 1.000 | 0.108 |
| Infective Complications | 19.4% (435/2247) | 14.2% (383/2704) | 7.4% (120/1617) | **<0.001** | **<0.001** | **<0.001** | **<0.001** |
| Urological Complications | 5.9% (132/2247) | 8.3% (224/2704) | 4.1% (66/1617) | **<0.001** | **0.003** | **0.038** | **<0.001** |
| Return to Theatre | 12.0% (269/2247) | 15.7% (424/2704) | 12.9% (208/1617) | **<0.001** | **<0.001** | 1.000 | **0.033** |
| 30-day Readmission** | 11.5% (249/2168)** | 10.2% (275/2704) | 14.4% (233/1617) | **<0.001** | 0.423 | **0.022** | **<0.001** |
| 30-day Mortality | 3.2% (71/2247) | 2.4% (65/2704) | 1.7% (27/1617) | **0.013** | 0.315 | **0.011** | 0.318 |
| 90-day Mortality | 4.5% (100/2247) | 4.5% (121/2704) | - | 0.967 | *NA* | *NA* | *NA* |

*Overall p-Values are from Chi-square tests, with pairwise comparisons from Bonferroni-corrected Chi-square tests unless stated otherwise. Bold p-values are significant at p<0.05. NA=pairwise comparisons are not applicable, as data are only available for two cohorts.AL=anastomotic leak, CN=conduit necrosis. *p-Values from Kruskal-Wallis tests (Overall) or Bonferroni-corrected Mann-Whitney tests (Pairwise), as the factor is ordinal. **The OGAA data excludes N=79 who either died in hospital, or where follow up was not available.*
